# Comparison of analgesic efficacy between rectus sheath blockade, intrathecal morphine with bupivacaine, and intravenous patient-controlled analgesia in patients undergoing robot-assisted laparoscopic prostatectomy: a prospective, observational clinical study

**DOI:** 10.1186/s12871-020-01208-2

**Published:** 2020-11-23

**Authors:** Jung-Woo Shim, Yun Jeong Cho, Minhee Kim, Sang Hyun Hong, Hyong Woo Moon, Sung Hoo Hong, Min Suk Chae

**Affiliations:** 1 Department of Anesthesiology and Pain medicine, Seoul St. Mary’s Hospital, College of Medicine, The Catholic University of Korea, 222, Banpo-daero, Seocho-gu, Seoul, 06591 Republic of Korea; 2 Department of Anesthesiology and Pain medicine, Bucheon St. Mary’s Hospital, College of Medicine, The Catholic University of Korea, Seoul, Republic of Korea; 3 Department of Urology, Seoul St. Mary’s Hospital, College of Medicine, The Catholic University of Korea, Seoul, Republic of Korea

**Keywords:** Rectus sheath bupivacaine, Intrathecal morphine with bupivacaine, Robot-assisted laparoscopic prostatectomy

## Abstract

**Background:**

We explored the analgesic outcomes on postoperative day (POD) 1 in patients undergoing robot-assisted laparoscopic prostatectomy (RALP) who received intravenous patient-controlled analgesia (IV-PCA), rectus sheath bupivacaine block (RSB), or intrathecal morphine with bupivacaine block (ITMB).

**Methods:**

This was a prospective, observational clinical trial. Patients were divided into three groups: IV-PCA (*n* = 30), RSB (*n* = 30), and ITMB (*n* = 30). Peak pain scores at rest and with coughing, cumulative IV-PCA drug consumption, the need for IV rescue opioids, and Quality of Recovery-15 (QoR-15) questionnaire scores collected on POD 1 were compared among the groups.

**Results:**

The preoperative and intraoperative findings were comparable among the groups; the ITMB group required the least remifentanil of all groups. During POD 1, the ITMB group reported lower levels of pain at rest and with coughing, compared with the other two groups. During POD 1, incidences of severe pain at rest (10.0% vs. 23.3% vs. 40.0%) and with coughing (16.7% vs. 36.7% vs. 66.7%) were the lowest in the ITMB group compared with the RSB and IV-PCA groups, respectively. After adjustment for age, body mass index, diabetes mellitus, hypertension, and intraoperative remifentanil infusion, severe pain at rest was 0.167-fold less common in the ITMB group than in the IV-PCA group, while pain with coughing was 0.1-fold lower in the ITMB group and 0.306-fold lower in the RSB group, compared with the IV-PCA group. The ITMB group required lower cumulative IV-PCA drug infusions and less IV rescue opioids, while exhibiting a better QoR-15 global score, compared with the other two groups. Complications (nausea and pruritus) were significantly more common in the ITMB group than in the other two groups; however, we noted no ITMB- or RSB-related anesthetic complications (respiratory depression, post-dural headache, nerve injury, or puncture site hematoma or infection), and all patients were assessed as Clavien-Dindo grade I or II during the hospital stay.

**Conclusion:**

Although ITMB induced complications of nausea and pruritus, this analgesic technique provided appropriate pain relief that enhanced patient perception related to early postoperative recovery.

**Trial registration:**

Clinical Research Information Service, Republic of Korea, (approval number: KCT0005040) on May 20, 2020

## Background

Robot-assisted laparoscopic prostatectomy (RALP) is a technically advanced, minimally invasive surgical method that affords much better surgical view and greater maneuverability than open or laparoscopic prostatectomy [1]. Previous studies have found that RALP is associated with better oncological and functional outcomes, compared with open or laparoscopic radical prostatectomy [2, 3]. However, RALP patients frequently experience unbearable pain that persists over several days after surgery and requires pain-relief medications, such as opioids. This pain arises from skin-port incisions, multiple dissections of prostate-involved and surrounding tissues, bladder spasm, and transurethral catheter irritation [4]. Various central and/or peripheral pain-relief methods have been used to attenuate the severe pain that develops immediately after RALP [5, 6]. A rectus sheath block (RSB) regimen affords peri-umbilical incision site analgesia superior to that achieved via local anesthetic infiltration; this site is the principal source of pain immediately after laparoscopy-based surgery [7]. Compared with transversus abdominis plane (TAP) block, RSB may afford better analgesia when a midline incision is created and more prolonged blockade of noxious input from that site [8]. An intrathecal morphine and bupivacaine block (ITMB) regimen affords pain relief for 20–48 h postoperatively and reduces bladder spasm-related discomfort (a common complication associated with urinary catheter insertion after prostate surgery) [9]. However, no ideal analgesic regimen has been described that affords maximal benefits with minimal side effects; this would improve the quality of early postoperative recovery after RALP.

The aim of this study was to evaluate the analgesic outcomes on postoperative day (POD) 1 in patients undergoing RALP who received RSB or ITMB, compared with patients who received intravenous patient-controlled analgesia (IV-PCA) alone. We also compared postoperative complications and patient satisfaction. We hypothesized that, due to a reduction in surgical pain and bladder spasm-related discomfort, the ITMB regimen would lead to improved pain relief and satisfaction with recovery on the first day after surgery, compared with the other analgesic regimens.

## Methods

### Ethical considerations

**Fig. 1:**
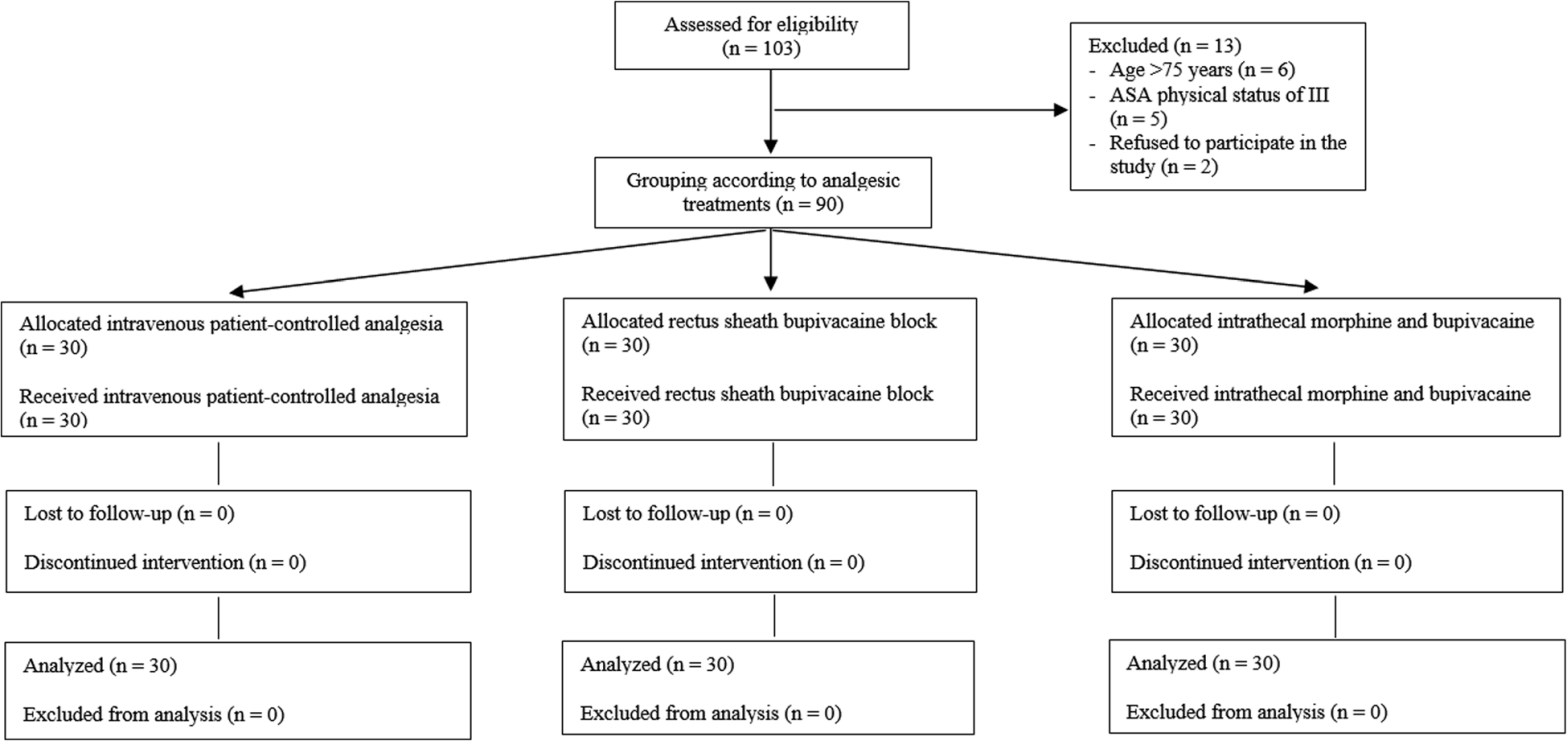
Flow chart of the study

This was a prospective, observational parallel-cohort trial. The protocol was approved by the Institutional Review Board of Seoul St. Mary’s Hospital Ethics Committee (approval no. KC20OISI0124) on April 29, 2020. The study was performed in accordance with all relevant principles of the Declaration of Helsinki. The study protocol was prospectively registered on a publicly accessible clinical registration site recognized by the International Committee of Medical Journal Editors (Clinical Research Information Service, Republic of Korea; approval no. KCT0005040) on May 20, 2020. Written informed consent was obtained from all patients enrolled between May 2020 and July 2020. The study adhered to Strengthening the Reporting of Observational Studies in Epidemiology guidelines (Additional file 1); a study flow chart is shown in Fig. 1.

### Study population

The inclusion criteria for our study were: age 19–74 years, prostate cancer stage I or II [10], patients scheduled for elective RALP, and American Society of Anesthesiologists (ASA) physical status I or II. The exclusion criteria were: a history of allergy to a local anesthetic or opioid drug, coagulopathy (international normalized ratio [INR] > 1.5 for ITMB or INR > 2.0 for a single injection of ultrasound-guided RSB; and platelet count < 100.0 ×  10^9^/L) [11, 12], hemodynamic instability that required strong vasopressors (i.e., epinephrine or norepinephrine), hetastarch colloid infusion, or blood product transfusion (i.e., packed red blood cells ≥1 unit due to hemoglobin < 7.0 g/dL) [13], and refusal to participate.

The patients were divided into three groups based on their analgesia preference: IV-PCA alone (reference group), RSB and IV-PCA (RSB group), and ITMB and IV-PCA (ITMB group).

### Patient management in the operating room

The RALP surgical technique and balanced anesthetic management were as described previously [14]; patient care was standardized apart from the analgesic treatments. Briefly, balanced anesthesia was performed by attending expert anesthesiologists. Induction of anesthesia was achieved using 1–2 mg/kg of propofol and 0.6 mg/kg of rocuronium; anesthesia was then maintained using 2.0–6.0% desflurane under medical air in oxygen. Remifentanil was continuously infused at a rate of 0.1–0.5 μg/kg/min, as appropriate. The Bispectral Index™ instrument was set between 40 and 50 to ensure appropriate hypnotic depth. Rocuronium was repeatedly infused under train-of-four monitoring (> 1 twitch). End-tidal CO_2_ was set between 30 and 40 mmHg with adjustment of the ventilator mode. For fluid therapy, a baseline isotonic crystalloid was prepared based on the estimated fluid maintenance requirements, which were established in accordance with the patient’s weight and anticipated tissue trauma. Additional fluid boluses were infused according to blood loss; however, the total amount of fluid was restricted to a maximum of 1 L before vesicourethral anastomosis.

**Fig. 2:**
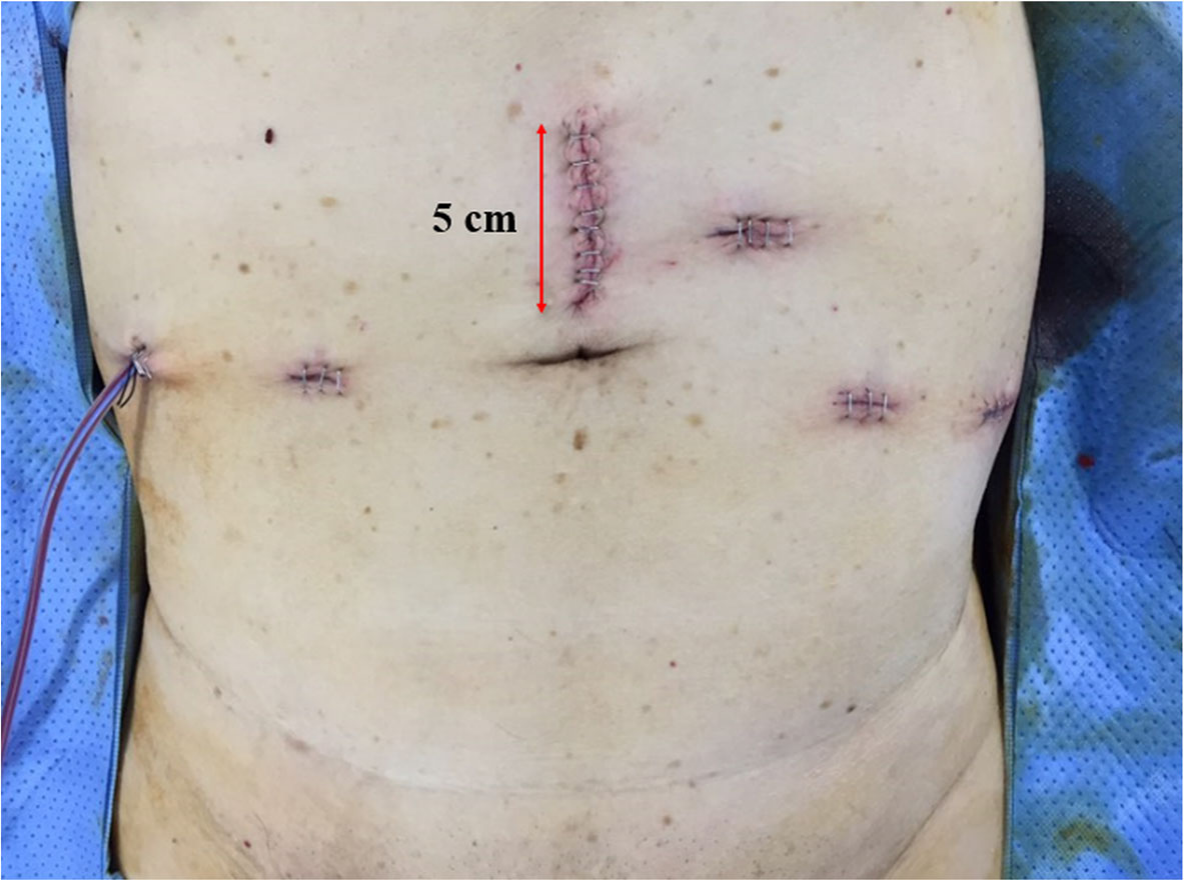
Peri-umbilical wound site (arrow) that is the principal analgesic target of RSB

The attending anesthesiologists (whose subspecialty involved regional blocks) and nurses were aware of the group allocations, but were not involved in later patient care or data collection (other than the completion of medical records). RSB was established immediately after the induction of general anesthesia. An ultrasound probe was positioned transversely on the rectus abdominis muscle, above the umbilicus (Fig. 2). Guided by real-time ultrasound, a sterile 22-G Tuohy-type epidural needle was cautiously advanced in-plane (to prevent injury to nearby vessels) from medially to laterally until the tip attained the plane between the lateral side of the rectus abdominis muscle and the posterior rectus sheath. After negative pressure aspiration, 20 mL of 0.25% (w/v) bupivacaine was administered and the block was repeated on the opposite side. ITMB was placed before the induction of general anesthesia. Each patient received 0.2 mg of intrathecal morphine sulfate and 7.5 mg of bupivacaine by means of a sterile 25-G Quincke-type spinal needle inserted between lumbar vertebrae 3 and 4. The drugs were administered through a single injection after collection of cerebrospinal fluid. All patients were allowed access to IV-PCA (1000 μg of fentanyl, 90 mg of ketorolac, and 0.3 mg of ramosetron). The IV-PCA regimen featured a 2-mL bolus injection and 0.5 mL/h basal infusion with a lockout time of 10 min. If a patient experienced acute postoperative breakthrough pain (visual analog scale [VAS] score ≥ 7), 25 mg of pethidine (an IV rescue opioid) was administered based on the discretion of the attending physicians (in the postoperative acute care unit or ward), who were blinded to group assignment.

### Pain outcomes

Cumulative IV-PCA drug consumption and the need for IV rescue opioids were primarily assessed during the first 24 h postoperatively. Peak pain scores at rest and with coughing were assessed using a VAS that ranged from 0 to 10, where “0” represented no pain and “10” represented the worst possible pain. Pain severity was classified as mild (VAS scores 0–3), moderate (4–6), or severe (7–10) [15]. Pain was assessed using the VAS three times (i.e., at 1 h postoperatively in the post-anesthesia care unit [PACU], as well as at 6 and 24 h postoperatively in the ward). If a patient experienced acute postoperative breakthrough pain (VAS score ≥ 7), 25 mg of pethidine (an IV rescue opioid) was administered by attending physicians (anesthesiologists in the PACU and urologists in the ward) or nurses, none of whom were aware of the group allocations.

### Clinical variables

Preoperative demographic and laboratory parameters were recorded on the day before surgery by attending urologic physicians or nurses in the ward, who were not aware of the group allocations and were not involved in further data collection other than filling in medical record forms. Intraoperative findings, such as surgical duration, hypotension status (systolic blood pressure < 90 mmHg for more than 10 min), total rescue ephedrine infusion, total remifentanil infusion, crystalloid fluid infusion, urine output, and hemorrhage status, were recorded by the attending anesthesiologists or nurses in the operating room, who were not involved in further patient care or data collection (other than filling in anesthetic record forms) and were not aware of the group allocations. Postoperative findings, such as the global quality-of-recovery score on a 15-item questionnaire (the QoR-15) [16]; the incidences of nausea, vomiting, and pruritus; the Clavien-Dindo grade [17]; and laboratory variables, were measured in the ward on the first day after surgery (between 6 and 8 pm). These findings were measured by anesthesiology residents (Y.J.C. and M.K.) who were not aware of the group allocations and were not involved in further patient care or data collection. Additionally, nausea and vomiting were assessed on a binary scale (yes/no). Patients were considered to have nausea, if they responded positively to the question, “are you or have you felt nauseated after surgery?”. Using similar questions, vomiting episodes were assessed [18]. Pruritus was assessed using the following scale: 0 = no itch; 1 = itch with no need to scratch, just rubbing (mild); 2 = itch with need to scratch (moderate); 3 = itch with need to scratch and requiring treatment (severe) [19]. We defined overt pruritus as a score ≥ 2. Therefore, nausea, vomiting, and pruritus were considered binary variables in our analysis.

### Statistical analyses

The minimum sample size was based on the difference in cumulative IV-PCA drug consumption on POD 1 between patients who received RSB and those who received ITMB, calculated using electronic medical records. Mean cumulative IV-PCA drug consumption on POD 1 by patients who received RSB (*n* = 10) and those who received ITMB (*n* = 10) were 47.1 and 29.1 mL, respectively. The standard deviation (SD) among the 20 patients was 23.6 mL. Therefore, a minimum sample size of 27 patients/group was required to afford an α value of 0.05 and a power of 0.8. We recruited 30 patients for each group; we assumed a dropout rate of 10%.

Data are expressed as means ± standard deviations (SDs), medians with interquartile ranges (IQRs), or numbers with proportions (%), as appropriate. The normality of continuous data distributions was evaluated using the Shapiro–Wilk test. Continuous perioperative variables of the three groups were compared via one-way analysis of variance or the Kruskal–Wallis test; post hoc testing employed the unpaired *t*-test or the Mann–Whitney U test. Perioperative categorical variables were compared among the groups using the Pearson *χ*^2^ test or Fisher’s exact test, as appropriate. Trend testing employed a linear-by-linear association method. To determine the clinical analgesic efficacy of the treatments, logistic regression analysis was used to derive odds ratios with 95% confidence intervals of the risks (postoperative peak VAS score ≥ 7 at rest and with coughing) associated with IV-PCA alone (reference), and the RSB and ITMB, after adjusting for age, body mass index, and diabetes mellitus and hypertension statuses (these comorbidities may change in accordance with pain level) [20, 21], and intraoperative remifentanil consumption. Tests for linear trends among patients in terms of cumulative IV-PCA drug consumption on POD 1 were based on stepwise linear regression. All tests were two-sided and a *p*-value < 0.017 was considered statistically significant (multiple comparisons were made). All statistical analyses were performed with the aid of SPSS for Windows (ver. 24.0; IBM Corp., Armonk, NY, USA) and MedCalc for Windows (ver. 11.0; MedCalc Software, Ostend, Belgium).

## Results

### Study population

In total, 103 patients were assessed for eligibility. Thirteen patients were excluded: six were aged > 74 years, five had ASA physical status III, and two refused to participate. Thus, 90 patients were enrolled and divided into the IV-PCA, RSB, and ITMB groups (*n* = 30 patients per group).

### Preoperative and intraoperative findings

Of all patients (*n* = 90), the median age was 65 (62–71) years and the median body mass index was 24.0 (22.5–26.5) kg/m^2^. In total, 15 patients (16.7%) had diabetes mellitus and 35 (38.9%) hypertension. None of the patients showed coagulopathic findings preoperatively; the minimum and maximum INRs were 0.8 and 1.0, while the minimum and maximum platelet counts were 120.0 × 10^9^/L and 355.0 × 10^9^/L. Table 1 shows that the preoperative and intraoperative findings were comparable among the three groups. However, during surgery, the ITMB group exhibited the lowest remifentanil consumption, whereas the RSB group required less remifentanil than the IV-PCA group.

**Table 1:** Comparisons of preoperative and intraoperative findings between the three groups

Group	IV-PCA	RSB	ITMB	
n	30	30	30	***p***
***Preoperative findings***				
Age (years)	65 (61–69)	67 (64–72)	65 (62–71)	0.286
Body mass index (kg/m^2^)	24.4 (22.7–27.7)	23.6 (22.2–25.2)	24.2 (22.3–26.4)	0.276
*Comorbidity*				
Hypertension	9 (30.0%)	10 (33.3%)	16 (53.3%)	0.134
Diabetes mellitus	7 (23.3%)	3 (10.0%)	5 (16.7%)	0.383
History of abdominal surgery	7 (23.3%)	4 (13.3%)	6 (20.0%)	0.602
*Laboratory variables*				
White blood cell count (× 10^9^/L)	5.4 (4.6–7.4)	7.1 (5.9–7.6)	6.2 (5.2–7.5)	0.133
Neutrophil (%)	54.3 (47.9–57.2)	55.1 (51.6–59.6)	52.9 (51.1–54.5)	0.118
Lymphocyte (%)	33.8 (31.3–38.3)	32.6 (31.1–40.4)	36.5 (34.6–39.0)	0.089
Hemoglobin (g/dL)	14.3 (13.8–14.8)	13.9 (13.1–15.4)	14.7 (13.7–15.6)	0.375
Platelet count (× 10^9^/L)	187.0 (160.5–221.0)	193.0 (169.8–242.0)	207.0 (172.3–232.3)	0.52
International normalized ratio	0.9 (0.8–0.9)	0.9 (0.9–0.9)	0.9 (0.8–0.9)	0.148
***Intraoperative findings***				
Surgical duration (min)	123 (109–145)	123 (100–141)	123 (114–138)	0.713
Hypotension event^a^	10 (33.3%)	15 (50.0%)	17 (56.7%)	0.175
Total rescue ephedrine infusion (mg)	0 (0–4)	2 (0–8)	4 (0–8)	0.139
Total remifentanil infusion (mg)	0.5 (0.4–0.6)	0.4 (0.3–0.4)^*^	0.2 (0.1–0.3)^*,†^	< 0.001
Crystalloid fluid infusion (mL)	500 (400–600)	575 (400–663)	525 (388–800)	0.782
Urine output (mL)	100 (50–100)	50 (50–100)	100 (50–100)	0.496
Hemorrhage (mL)	100 (50–100)	100 (50–100)	100 (50–163)	0.405

Abbreviations: *IV-PCA* Intravenous patient-controlled analgesia, *VAS* Visual analog scale, *PACU* Post-anesthesia care unit

^*^p < 0.017 as statistical significance based on the level in the IV-PCA group

^†^*p* < 0.017 as statistical significance based on the level in the RSB group

^a^Hypotension event defined as systolic blood pressure < 90 mmHg over 10 min

NOTE: Values are expressed as the median (interquartile) and number (proportion)

### Postoperative pain

**Fig. 3:**
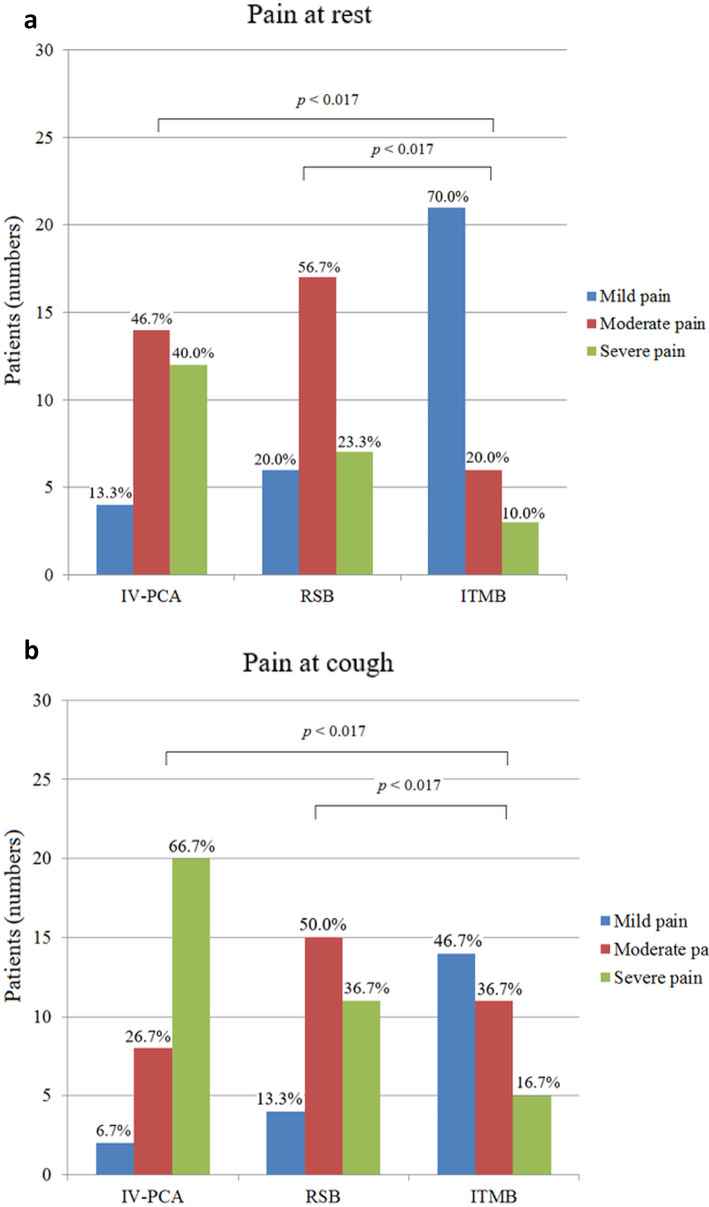
Pain scores (**a**) at rest and (**b**) with coughing in the three groups (*n* = 30 per group) in the first 24 h postoperatively. Mild pain was defined as a peak VAS score of 0–3, moderate pain as a peak score of 4–6, and severe pain as a peak score of 7–10. *p* < 0.017 indicates statistical significance (adjusted for multiple comparisons). Values are expressed as numbers with proportions (% values)

During POD 1, the ITMB group reported lower pain levels at rest and with coughing than did the RSB and IV-PCA groups (Fig. 3). After adjustment for age, body mass index, comorbidity status, and intraoperative remifentanil infusion, severe pain at rest was 0.167-fold less common in the ITMB group than in the IV-PCA group, while pain with coughing was 0.1-fold lower in the ITMB and 0.306-fold lower in the RSB group, compared with the IV-PCA group (Table 2). Table 3 shows that cumulative IV-PCA drug consumption decreased according to analgesic treatment in the following order: IV-PCA alone > RSB > ITMB (linear regression, *p* < 0.001). The ITMB group required less IV-PCA drug infusion and IV rescue opioids than did the RSB and IV-PCA groups. The ITMB group had the lowest peak VAS scores at rest and with coughing, compared with the other two groups, while the RSB group had a lower peak VAS score with coughing, compared with the IV-PCA group (Table 4).

**Table 2:** Analgesic efficacy of IV-PCA, the RSB, and ITMB block with severe pain (peak VAS ≥7) at rest and cough during 24 h postoperatively

	***ß***	Odds ratio	95% Confidence interval	***p***
***Severe pain at rest***				
Analgesia adjusted for age, BMI, DM, hypertension, and intraoperative remifentanil consumption				
IV-PCA	Reference			
RSB	−0.74	0.477	0.156–1.464	0.196
ITMB	−1.792	0.167	0.041–0.675	0.012
***Severe pain at cough***				
Analgesia adjusted for age, BMI, DM, hypertension, and intraoperative remifentanil consumption				
IV-PCA	Reference			
RSB	−1.186	0.306	0.105–0.888	0.029
ITMB	−2.303	0.1	0.029–0.34	< 0.001

Abbreviation: *VAS* Visual analog scale, *IV-PCA* Intravenous patient-controlled analgesia, *RSB* Rectus sheath block, *ITMB* Intrathecal morphine with bupivacaine, *BMI* Body mass index, *DM* Diabetes mellitus

**Table 3:** Linear trend for cumulative IV-PCA drug consumption during POD 1 among the IV-PCA vs. RSB vs. ITMB groups

	***ß***	95% Confidence interval	***p***
***Cumulative IV-PCA drug consumption during POD 1 (mL)***			
Adjusted for age, BMI, DM, hypertension, and intraoperative remifentanil consumption			
Linear trend among the IV-PCA vs. RSB vs. ITMB groups	−10.715	− 16.403 - -5.026	< 0.001

Abbreviation: *IV-PCA* Intravenous patient-controlled analgesia, *POD* Postoperative day, *RSB* Rectus sheath block, *ITMB* Intrathecal morphine with bupivacaine, *BMI* Body mass index, *DM* Diabetes mellitus

**Table 4:** Comparisons of patient outcomes during the first 24 h postoperatively between the three groups

Group	IV-PCA	RSB	ITMB	
n	30	30	30	***p***
*Requirement of opioid infusion*				
Cumulative IV-PCA infusion (mL)	37.6 (25.9–57.3)	42.8 (29.9–60.8)	18.7 (14.7–26.2)^*, †^	< 0.001
IV rescue opioid infusion	19 (63.3%)	21 (70.0%)	6 (20.0%)^*, †, ‡^	< 0.001
Peak visual analog scale				
at rest	6 (4–7)	5 (4–6)	3 (2–4)^*, †^	< 0.001
at cough	8 (6–9)	6 (5–7)^*^	4 (3–5)^*, †^	< 0.001
*Quality of early recovery*				
Global score of QoR-15 questionnaire on POD 1	124 (122–129)	124 (117–133)	130 (126–141)^*,†^	0.002
*Complications*				
Nausea	2 (6.7%)	2 (6.7%)	9 (30.0%)^‡^	0.012
Vomiting	0 (0.0%)	1 (3.3%)	2 (6.7%)	0.355
Pruritus	0 (0.0%)	0 (0.0%)	5 (16.7%)^‡^	0.005
*Laboratory variables on POD 1*				
White blood cell count (× 10^9^/L)	13.5 (8.7–16.5)	15.4 (8.2–20.7)	14.3 (11.2–19.3)	0.478
Neutrophil (%)	172.0 (141.0–203.3)	175.5 (154.0–211.8)	168.0 (151.3–201.0)	0.873
Lymphocyte (%)	73.5 (69.1–76.5)	73.1 (68.0–78.7)	71.4 (65.1–78.9)	0.807
Hemoglobin (g/dL)	8.8 (7.4–11.0)	7.8 (7.1–9.1)	8.7 (6.6–10.3)	0.476
Platelet count (× 10^9^/L)	12.6 (11.7–13.4)	12.2 (11.0–13.5)	12.3 (11.7–12.9)	0.419
International normalized ratio	1.0 (0.8–1.0)	0.9 (0.9–1.0)	0.9 (0.8–1.0)	0.072

Abbreviations: *QoR-15* Quality of Recovery-15 questionnaire, *POD* Postoperative day

^*^p < 0.017 as statistical significance based on the level in the IV-PCA group

^†^*p* < 0.017 as statistical significance based on the level in the RSB group

^‡^*p* < 0.05 using the linear-by-linear method

NOTE: Values are expressed as the median (interquartile) and number (proportion)

### Postoperative clinical findings

The global QoR-15 questionnaire score was higher in the ITMB group than in the RSB and IV-PCA groups (Table 4). Complications (nausea and pruritus) were significantly more common in the ITMB group than in the other two groups; however, we noted no ITMB- or RSB-related anesthetic complications (respiratory depression, post-dural headache, nerve injury, or puncture site hematoma or infection), and all patients were assessed as Clavien-Dindo grade I or II during the hospital stay.

## Discussion

Our principal findings were that ITMB may afford superior analgesia and better patient perception of outcome in terms of early postoperative recovery, compared with RSB or IV-PCA alone, in patients undergoing RALP. The analgesic efficacy of ITMB was approximately three-fold and 10-fold better in terms of reducing severe pain during the early postoperative period, compared with RSB and IV-PCA alone, respectively. Although ITMB was associated with more nausea and pruritus than RSB and IV-PCA alone, we noted no ITMB-related, postoperative adverse event, such as respiratory depression, lower leg numbness, or post-dural puncture headache.

Our ITMB data are similar to those of previous laparoscopic and open surgery reports [5, 22–24]; thus, ITMB is a feasible and practicable form of pain relief (yielding a lower pain score and lower opioid requirement). Moreover, it was not associated with serious complications (such as nerve injury) during or after surgery, and was better than RSB or IV-PCA alone. The differences between intrathecal and peripheral blocks include the sites affected by the analgesic drugs and subsequent drug actions. Intrathecally injected morphine and bupivacaine become widely dispersed in cerebrospinal fluid, thus more reliably (compared with RSB) preventing nociceptive inputs from multiple somatic dermatome levels in patients undergoing RALP [24, 25]. The principal skin wound created during laparoscopy-based surgery lies in the peri-umbilical area; the orifice is used for camera insertion and specimen (prostate mass) removal. In the past, RSB effectively countered pain caused by injury to the peri-umbilical dermatomes [26, 27]. However, as surgery advanced from open surgery to human-executed laparoscopic surgery to RALP (reducing the operation time and numbers of painful stimuli delivered by surgical wounds in sites such as the umbilicus) [28], the analgesic effect of RSB seems to have gradually decreased along with improvements in surgical wound care techniques. Furthermore, ITMB may deliver visceral analgesia by interacting with spinal μ- and ĸ-opioid receptors and voltage-gated sodium channels that contain binding sites for local anesthetics. It is now possible to totally (and simultaneously) avoid the surgical stress and pain imparted by intra-abdominal wounds (created when prostate-adjacent tissues are dissected and retracted) and skin wounds (created when the skin is incised, punctured, and retracted) [29, 30]. However, RSB inhibits only somatic, afferent nerve pain. Thus, it cannot deliver comprehensive analgesia; pain from the visceral origins is not dulled [26].

In terms of complications, postoperative nausea/vomiting and pruritus compromise the quality of patient recovery [31]. Previous studies suggested that the incidences of such complications were higher in patients who received intrathecal morphine than in those receiving local anesthetic-based analgesia [32–34]. Intrathecal block with low doses of combined morphine (75 or 100 μg) and bupivacaine (5 mg) provided more effective postoperative analgesia than did sham subcutaneous block with normal saline. However, the side effects (e.g., nausea and vomiting) were of comparable incidence and severity between the two groups [35]. Our ITMB regimen included bupivacaine (7.5 mg), which allowed us to reduce the morphine dose to 0.2 mg, thus reducing nausea/vomiting and pruritus while maintaining appropriate analgesia. However, we found higher incidences of nausea and pruritus in patients who received ITMB than in those who received RSB or IV-PCA alone. Notably, our incidences of nausea (30.0%), vomiting (6.7%), and pruritus (16.7%) in the ITMB group may be lower than those of patients who receive intrathecal morphine (without additive bupivacaine). Specifically, the reported incidences of nausea/vomiting and pruritus after surgery were approximately 60–80% and 30–100%, respectively [36–38]. Nguyen et al. [39] suggested that the addition of bupivacaine (15 mg) to intrathecal morphine (0.4 mg) improved pain relief and reduced the incidence of adverse events (such as hypotension) in patients undergoing laparoscopic liver resection. Girgin et al. [40] found that the incidence of pruritus increased as the dose of intrathecal morphine rose from 0.1 to 0.4 mg; however, when morphine was combined with low-dose bupivacaine (7.5 mg), the complication rate was reduced while analgesia remained stable in women undergoing cesarean sections. The good analgesia (i.e., reduced requirement for IV opioid infusion) without adverse complications (Clavien-Dindo grade ≥ III), afforded by our low-dose ITMB regimen, may enhance early postoperative recovery compared with patients treated via RSB or IV-PCA alone.

In our study, there were comparable clinical characteristics, such as surgical duration, hypotension events, and total rescue ephedrine infusion, between the groups. Notably, analgesic treatments (RSB vs. ITMB) may not affect the prolongation of surgical time that possibly results from management by attending anesthesiologists who have excellent technique and sufficient experience with regional blocks, although there were differences in the RSB and ITMB regimens, such as target sites (rectus muscle vs. spine), needle manipulation (ultrasound-based vs. palpation-based), and patient position (supine vs. lateral) [41, 42]. Furthermore, intrathecal injection of bupivacaine may produce a high level of sensory and motor block, as well as arterial hypotension. However, these effects were dose-dependent, such that the lowest dose of bupivacaine (7 mg) provided equally rapid onset and effective anesthesia for cesarean surgery while reducing the incidence of hypotension, compared with bupivacaine doses of 8 and 9 mg, in patients who received combined administration of intrathecal morphine (100 μg) [43]. These findings were consistent with ours in that a low dose of additive intrathecal bupivacaine (7.5 mg) may have minimal impact on the occurrence of persistent hypotension and requirement for rescue inotrope.

Our work had certain limitations. First, we delivered single bupivacaine injections to the rectus sheath when comparing the outcomes of the three pain-relief regimens. However, no ideal regional analgesic technique for RALP has yet been established; other regional analgesic models, including catheter-delivered continuous blockade, may be superior to ITMB [44]. Second, because of the absence of robust evidence related to an acceptable range of INRs for single-injection ultrasound-guided subfascial block (i.e., RSB), we presumed that an INR > 2.0 was the highest acceptable level for inclusion in our study. However, because of the risk of hematoma, skillful and meticulous ultrasound-based RSB is required for patient safety and analgesic results. Further RSB analyses are needed to determine the acceptable ranges of coagulopathic parameters, such as the INR. Third, our study was limited in that patients were not randomly allocated, despite the presence of comparable groups. A randomized setting was considered but rejected due to ethical concerns that IV-PCA alone may provide insufficient pain relief, compared with the other two analgesic regimens. Therefore, it was not possible to determine whether the analgesic results were solely related to pain-relief regimens.

## Conclusions

ITMB may usefully reduce postoperative pain and aid recovery in patients undergoing RALP. Although robot-assisted surgery is more advanced and less invasive than open or laparoscopic surgery, analgesic care must counter both parietal and visceral pain associated with multi-level skin wounds and intra-abdominal tissue injuries. Our ITMB regimen (a low dose of morphine [0.2 mg] combined with bupivacaine [7.5 mg]) may contribute superior analgesia and better patient perception in terms of early postoperative recovery.

## Supplementary Information

**Additional file 1.** “Strengthening the Reporting of Observational Studies in Epidemiology” (STROBE) guidelines.

## Data Availability

The datasets used and/or analyzed during this study are available from the corresponding author on reasonable request.

## Abbreviations

RALP: Robot-assisted laparoscopic prostatectomy
RSB: Rectus sheath block
TAP: Trasnversus abdominis plane block
ITMB: Intrathecal morphine and bupivacaine block
POD: Postoperative day
IV-PCA: Intravenous patient-controlled analgesia
ASA: American Society of Anesthesiologists
INR: Interntaional normalized ratio
VAS: Visual analogue scale
QoR-15: Quality-of-recovery score on a 15-item questionnaire
